# SOCIAL INTERACTIONS AND LONELINESS AMONG THE ADULT LIFESPAN: A COVID-19 COORDINATED ANALYSIS

**DOI:** 10.1093/geroni/igad104.0263

**Published:** 2023-12-21

**Authors:** Daisy Zavala, MacKenzie Hughes, Rita Hu, Sumbleen Ali, Reilly Kincaid, Destiny Ogle

**Affiliations:** Stony Brook University, Stony Brook, California, United States; Georgia Institute of Technology, Atlanta, Georgia, United States; University of Michigan, Ann Arbor, Michigan, United States; University of Connecticut, Storrs, Connecticut, United States; Purdue University, West Lafayette, Indiana, United States; Purdue University, West Lafayette, Indiana, United States

## Abstract

The COVID-19 pandemic included physical distancing mandates. Examining responses to isolation across the lifespan during the first year of the pandemic may shed light on age differences in social interactions and loneliness during a time of uncertainty. This coordinated analysis of 2 intensive longitudinal datasets (DCF, EAS) examines social interactions, loneliness, and age during the COVID-19 pandemic. It is a component of a larger, pre-registered coordinated analysis involving traditional longitudinal and cross-sectional studies (https://osf.io/jt4wf). DCF was a diary study of 221 adults ages 21-78 years (M = 48.92, SD = 14.81) who completed online questionnaires every day for 21 consecutive days. DCF participants were drawn from across the USA via MTurk and Qualtrics. Data collection spanned Oct-29-2020 to Nov-18-2020. EAS is an ongoing ecological momentary assessment measurement burst study; data from 82 adults aged 73-96 years (M = 79, SD = 5.41) who completed a 2 week burst of 4 EMA per day between Feb-1-2020 and July-1-2020 were used in this analysis. EAS participants were recruited from registered voter lists in Bronx, NY USA. Separate multilevel models (DCF: 2 level, EAS: 3 level) were conducted with the same covariates and constraints. Between and within individuals, social interactions did not predict loneliness in DCF; older age predicted lower loneliness. In EAS, individuals with more social interactions were less lonely, males reported higher levels of loneliness and relatively younger individuals had lower loneliness following interactions. Results illustrate the value of coordinated analysis for understanding loneliness under pandemic conditions.

